# The dengue preface to endemic in mainland China: the historical largest outbreak by *Aedes albopictus* in Guangzhou, 2014

**DOI:** 10.1186/s40249-017-0352-9

**Published:** 2017-09-22

**Authors:** Lei Luo, Li-Yun Jiang, Xin-Cai Xiao, Biao Di, Qin-Long Jing, Sheng-Yong Wang, Jin-Ling Tang, Ming Wang, Xiao-Ping Tang, Zhi-Cong Yang

**Affiliations:** 1 Guangzhou Center for Disease Control and Prevention, Guangzhou, 510440 People’s Republic of China; 2 Department of Epidemiology, Medical College, Jinan University, Guangzhou, 510632 People’s Republic of China; 3 Division of Epidemiology, The Jockey Club School of Public Health and Primary Care, The Chinese University of Hong Kong, Hong Kong, 999077 People’s Republic of China; 4 Department of Infectious Diseases, Guangzhou No. 8 People’s Hospital, Guangzhou, 510060 People’s Republic of China

**Keywords:** Dengue, Co-circulation, Dengue virus-1, Dengue virus-2, Endemic disease

## Abstract

**Background:**

Dengue was regarded as a mild epidemic in mainland China transmitted by *Aedes albopictus*. However, the 2014 record-breaking outbreak in Guangzhou could change the situation. In order to provide an early warning of epidemic trends and provide evidence for prevention and control strategies, we seek to characterize the 2014 outbreak through application of detailed cases and entomological data, as well as phylogenetic analysis of viral envelope (E) gene.

**Methods:**

We used case survey data identified through the Notifiable Infectious Disease Report System, entomological surveillance and population serosurvey, along with laboratory testing for IgM/IgG, NS1, and isolation of viral samples followed by E gene sequencing and phylogenetic analysis to examine the epidemiological and molecular characteristics of the outbreak.

**Results:**

The 2014 dengue outbreak in Guangzhou accounted for nearly 80% of total reported cases that year in mainland China; a total of 37,376 cases including 37,340 indigenous cases with incidence rate 2908.3 per million and 36 imported cases were reported in Guangzhou, with 14,055 hospitalized and 5 deaths. The epidemic lasted for 193 days from June 11 to December 21, with the highest incidence observed in domestic workers, the unemployed and retirees. The inapparent infection rate was 18.00% (135/750). In total, 96 dengue virus 1 (DENV-1) and 11 dengue virus 2 (DENV-2) strains were isolated. Phylogenetic analysis indicated that the DENV-1 strains were divided into genotype I and V, similar to the strains isolated in Guangzhou and Dongguan in 2013. The DENV-2 strains isolated were similar to those imported from Thailand on May 11 in 2014 and that imported from Indonesia in 2012.

**Conclusions:**

The 2014 dengue epidemic was confirmed to be the first co-circulation of DENV-1 and DENV-2 in Guangzhou. The DENV-1 strain was endemic, while the DENV-2 strain was imported, being efficiently transmitted by the *Aedes albopictus* vector species at levels as high as *Aedes aegypti*.

**Electronic supplementary material:**

The online version of this article (doi:10.1186/s40249-017-0352-9) contains supplementary material, which is available to authorized users.

## Multilingual abstracts

Please see Additional file 1 for translations of the abstract into the five official working languages of the United Nations.

## Background

Dengue is a mosquito-borne viral infection that can produce a wide spectrum of symptoms, from a mild febrile illness progressing to dengue hemorrhagic fever (DHF) and dengue shock syndrome (DSS). Over the past 50 years, dengue has affected more than 100 countries in tropical and subtropical areas accompanying a 30-fold increase in global incidence [1]. In recent years, the incidence and disease burden are increasing dramatically due to human population growth, spread of mosquito vectors, globalization, urbanization, and climate change [2, 3]. Estimates of new annual infections globally of 50–100 million from World Health Organization (WHO) have been suspected to be severe underestimates of actual incidence [4].

The dengue virus (DENV) is a member of the genus *Flavivirus* family Flaviviridae*,* and is a single-stranded positive-sense, RNA virus with a genome of about 11 kb. It is antigenically divided into four serotypes (DENV-1, DENV-2, DENV-3, and DENV-4). The four virus serotypes are transmitted by mosquito vector species *Aedes albopictus* and *Aedes aegypti* and circulate in tropic and subtropical areas, transmission being largely dominated by one strain in different regions and times [5]. A serotype-specific lifelong immunity is developed after initial infection, subsequent infection by another serotype however will promote the risk of DHF/DSS due to antibody-dependent enhancement [5]. Furthermore, there are still factors contributing to the challenge of vaccine development, such as viral interference, reversion to virulence, antibody-dependent enhancement (ADE) and the lack of immunologic correlates and good animal models [6].

In recent years, the geographical dispersion of dengue accelerated sharply mainly due to imported dengue cases. From 1999 to 2010 in Japan, the number of imported cases has increased annually with 868 total imported cases over that period according to the Infectious Diseases Control Law [7]. In 2014 Japan saw 160 confirmed indigenous cases [8], after 70 years with no confirmed autochthonous cases, with one case imported to England related to an outbreak in Yoyogi Park [9]. In Europe, the risk of transmission has increased from 2010 [10]. In 2013, Germany diagnosed one imported case from resident travel to Japan contracting DENV-2, however, no indigenous cases were reported in Japan [11]. Additionally, a DENV-3 patient was diagnosed in Germany in a traveler returning from West Africa [12]. After an absence of 55 years, it has re-emerged in Europe both as autochthonous sporadic cases and as an outbreak in Madeira [2]. The identification of autochthonous dengue cases in France in 2010, 2013, and 2014, indicated DENV reservoir had been mantained in the local *Aedes albopictus* vector species [13–15].

Guangzhou is one of largest city having the highest population density in the world. It is the capital city of Guangdong Province in southern China, located at 112°57 E to 114°3 E and 22°26 N to 23°56 N, with 10 administrative districts (Liwan, Yuexiu, Haizhu, Baiyun, Tianhe, Huangpu, Luogang, Panyu, Nansha, Huadu) and 2 satellite cities (Conghua and Zengcheng), covering 7434.40 km^2^ and with a current population of more than 12.84 million and a humid subtropical climate influenced by the Asian monsoon season [16]. In the past three decades, *Aedes albopictus* had been monitored as the vector for dengue transmission in Guangzhou, with no *Aedes aegypti* identified. It ranked second highest in proportion amongst all adult mosquitoes from surveillance at about 7% compared to 8% of *Culex fatigans* which took the largest proportion. Guangzhou is the most important city in China for annual DENV transmission, accounting for more than 50% of the DENV cases in mainland China [17]. DENV-1, DENV-2, DENV-3, and DENV-4 circulated sequentially in Guangzhou from initial identification in 1978 to 2013. Annual reported cases have been climbing, especially between 2010 and 2013, with reports of 59, 33, 139, and 1249 cases, respectively. Prior to the 2014 outbreak, 2013 was the largest dengue outbreak in the past decade. However, reported cases soared dramatically beyond academic and government expectation in 2014, accounting for approximately 80% of reported cases in mainland China. In order to recognize epidemic trend and provide evidence for prevention and control strategies, we investigated various data sources from the 2014 outbreak including epidemiologically relevant characteristics of cases, laboratory testing and phylogenetic analysis of E gene sequences from isolated DENV samples.

## Methods

### Case definition and reporting

According to the Diagnostic Criteria for Dengue Fever (WS216–2008) enacted by the Chinese Ministry of Health [18], a suspected case is confirmed if a patient presented with acute onset of fever (39–40 °C within 24–36 h), cephalalgia, arthralgia, myalgia, malaise, rash, accompanied by facial flushing, skin erythema, conjunctival congestion, and leukocytopenia, thrombocytopenia, or a positive tourniquet test. Clinically diagnosed cases were defined as suspected cases testing positive for IgM/IgG or nonstructural protein 1 (NS1) antigen by immune colloidal gold technique against DENV in serum or in patients whose residence was Guangzhou. Confirmed cases were distinguished if the diagnosed case had positive DENV RNA detected in serum by real-time fluorescent quantitative reverse transcription-PCR (qRT-PCR) or virus isolation or a four-fold increase of IgG titre in paired serum samples by Capture ELISA. Patients identified from passive surveillance after seeking medical attention or from active case surveillance conducted by the Center for Disease Control and Prevention (CDC) after initial dengue transmission occurs should be reported to the Notifiable Infectious Disease Reporting System (NIDRS) within 24 h.

### Survey methods

A standardized case questionnaire was applied during face-to-face interviews with patients, which included general individual information, progression of disease and treatment, symptoms, physical examination, clinical laboratory test and contact history. In addition, serum specimens of patients were obtained with ethical approval from the Ethics Committee of Guangzhou Center for Disease Control and Prevention (CDC) and written informed consent from all patients once who sought medical service. Additionally, samples from healthy persons for population serosurvey were obtained in the main outbreak communities. A main outbreak community was defined as a core area within 200 m around a case’s residence or workplace. In these areas, blood samples from people bitten by mosquitoes, significant outdoor exposure, or got fever were collected for dengue - specific IgM detection.

### Entomological surveillance

During the outbreak, mosquito density was monitored using *Breteau Index* (BI), *Standard Space Index* (SSI) and *Adult Mosquito Density Index* (ADI) by CDC. The BI measuring of indoors and SSI of outdoors are two conventional *Aedes* larval indices applied to evaluate mosquito density [19]. The index is calculated as follows: BI = number of positive containers per 100 houses, SSI = number of positive containers per 100 standard spaces. The ADI measurement of adult mosquitos was calculated by the number of *Aedes* collected per hour and per person by human landing catches.

Meanwhile, *Aedes albopictus* mosquitoes were collected around epidemic focal sites both indoors and outdoors. The larvae were reared to adulthood under standard laboratory conditions (28 ± 2 °C at 75–85% relative humidity). The emerging adults were maintained for 3–4 days, sorted by species and gender, pooled (50 individuals/pool), and then stored at −80°C [20].

### Laboratory methods

**Fig. 1:**
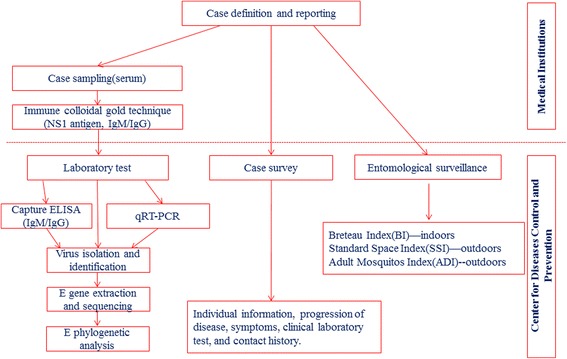
The survey and study technical route of the 2014 historical largest dengue outbreak in Guangzhou

See the summary of technical route in Fig. 1. In medical institutions, the rapid test by immune colloidal gold technique (ICGT) for preliminary screening was applied to detect NS1 antigen and IgM/IgG in serum samples from patients according to the manufacturer’s instructions. The diagnostic cassettes were from two companies, Wondfo (China) and Wantai (China) for NS1 antigen, and Panbio (Australia) for IgM/IgG.

After serum samples from suspected cases were sent to Guangzhou CDC, confirmatory tests were conducted by Capture ELISA, qRT-PCR, and viral isolation. Dengue IgM/IgG Capture ELISA kits were from Panbio (Australia) and qRT-PCR for DENV (serotypes 1–4) from Daan (China). The methods above were according to the manufacturer’s instructions respectively. The viruses were isolated by inoculating acute phase serum or mosquito samples into cell cultures from the mosquito, *Aedes albopitus*, clone C6/36 grown in 1640 medium (GIBCO, USA) with 2% fetal calf serum. After absorption at 28 °C for 1 h, C6/36 cells were incubated for 7 days at 28 °C. After 3 generations of passages, the CPE was verified by indirect immunofluorescence (IIF).

In the positive samples from viral isolation, RNA extraction and reverse transcriptase PCR (RT-PCR) of E gene were implemented followed by sequencing, assembly and phylogenetic analysis.

#### RNA extraction

Viral RNA was extracted using the QLAamp Viral RNA Mini Kit (Qiagen, Germany) from supernatant of infected cells according to the manufacturer’s instructions.

#### Primer design and RT-PCR

To amplify the entire sequence of the DENV E gene, DEN750 (5′-CAAGAACCGAAACGTGGATG-3′) and DEN2639 (5′-TGTGGAAGCAAATATCACCTG-3′) were designed for DENV-1, and DEN2F(5′-CCAGGCTTTACCATAATGGC-3′) and DEN2R(5′- CCAGCTGCACAACGCAACCAC-3′) were for DENV-2. RNA samples were used for one-step RT-PCR (TaKaRa, Japan). The RT-PCR reaction was performed at 50 °C for 30 min for reverse transcription, denaturation at 94 °C for 2 min, 35 cycles of denaturation at 94 °C for 30 s, primer annealing at 60 °C for 1.5 min, and extension at 72 °C for 3 min. A final extension at 72 °C for 10 min was performed to ensure complete double-stranded DNA synthesis. The PCR products were purified using QIA-quick PCR purification kits (Qiagen, Germany) following the manufacturer’s instructions [21].

#### Sequencing and assembly

The envelope genes were purified and completely sequenced using the BigDye Terminator Cycle Sequencing Kit (Applied Biosystems, CA) using previously described primers and following the manufacturer’s protocol. Sequences assemblies were completed using the SeqMan II software (DNASTAR, Inc., Madison, WI).

### Statistical analysis

Demographic, epidemiological and entomological data were analysed by R stats, plyr,ggplot2 and maptools packages (version 3.1.2, R Foundation for Statistical Computing, Vienna, Austria). The homologies of E gene nucleic acids were calculated and a phylogenetic tree was constructed using maximum likelihood (ML) method with a Kimura 2 parameter model using MEGA 5.0 software (http://www.megasoftware.net). 1000 bootstrap repetitions were applied to evaluate support of the consensus phylogenetic tree [21]. The 55 DENV-1 and 45 DENV-2 isolates used in this study were published in GenBank, representing a wide range of geographic spaces and time periods.

## Results

A total of 37,376 suspected cases were reported during the outbreak, about 2.4 times greater than the figure of 15,645 total identified cases from 1978 to 2013, including 37,340 autochthonous cases with incidence rate 2908.3 per million and 36 imported cases from abroad. There were 15,998 laboratory confirmed cases and 21,342 clinically diagnosed cases among the indigenous cases, with 14,055 (37.64%) hospitalized cases and 5 deaths.

### Epidemiological characteristics of autochthonous cases

#### Time distribution

**Fig. 2:**
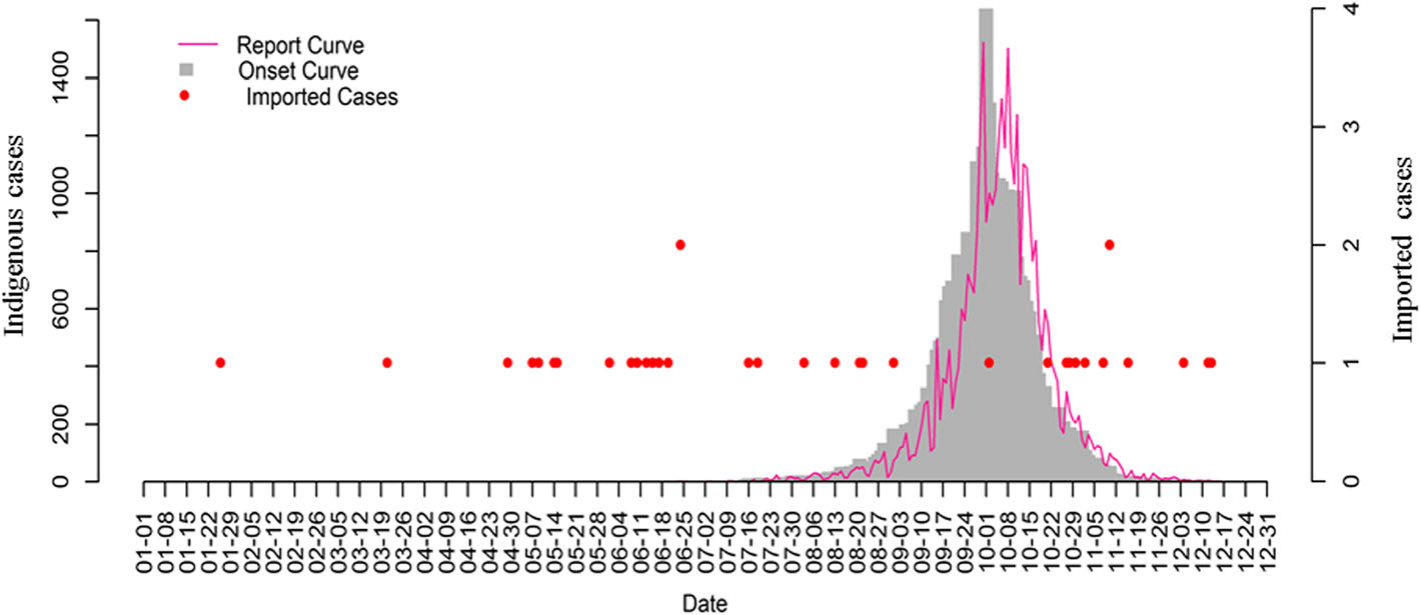
The daily onset and report curve of the 2014 historical dengue outbreak in Guangzhou. The grey histogram denotes the epicurve, the deep pink line shows the report time curve, and the red points depicts the daily number of imported cases from abroad

The epidemic period lasted for 193 days from June 11 to December 21, peaking at October with 18,557 (49.70%) cases. There were 15 (0.04%), 239 (0.64%), 1703 (4.56%), 15,626 (41.85%), 1159 (3.10%) and 41 (0.11%) cases in June, July, August, September, November and December, respectively (see the details in Fig 2).

#### Geographic distribution

**Fig. 3:**
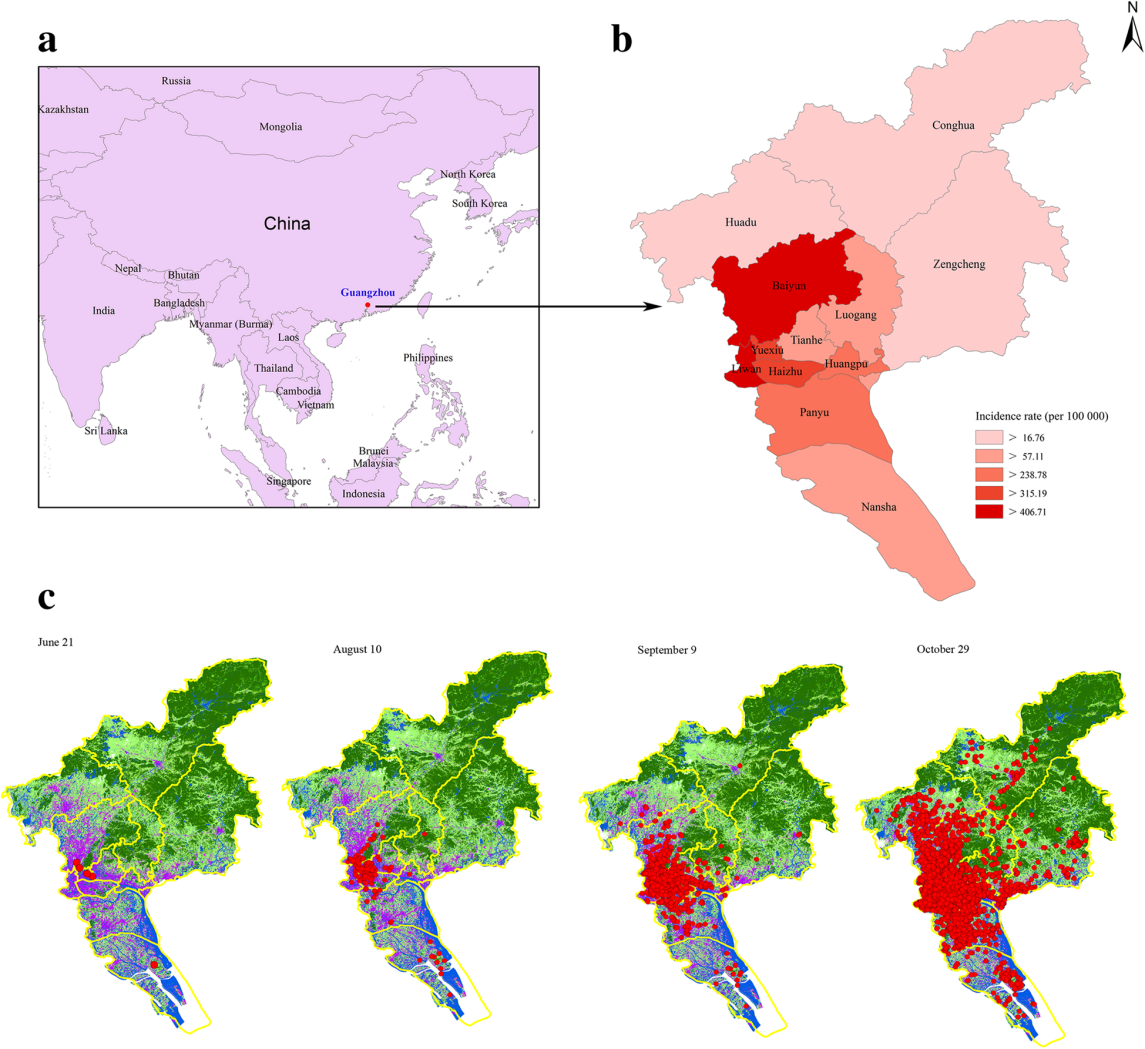
The geographic distribution of dengue outbreak in Guangzhou, 2014. **a** shows the location of Guangzhou city by red dot in China. **b** is the enlargement of Guangzhou, showing the incidence map of the epidemic. The more deepening the red color, the higher the incidence rate. **c** demonstrates the spatial evolution by case spot map in different time, including June 21, August 10, September 9, and October 29

As illustrated in Fig. 3 and Additional file 2: Table S1**,** all twelve districts of Guangzhou were affected by the outbreak. The highest incidence occurred in Baiyun with 530.47 per 100,000 (11,834 cases), followed by Liwan 488.68 per 100,000 (4466), Yuexiu 406.71 per 100,000 (4790), Haizhu 379.66 per 100,000 (5995), Huangpu 315.19 per 100,000 (1449), Panyu 248.73 per 100,000 (3540), Tianhe 238.78 per 100,000 (3431), Luogang 96.02 per 100,000 (359), Nansha 79.46 per 100,000 (483), Huadu 57.11 per 100,000 (542), Zengcheng 32.45 per 100,000 (348) and the lowest in Conghua 16.76 per 100,000 (103), respectively.

In total, 159 (96.95%) communities suffered cases during the epidemic, with 5 communities reporting no cases including Longxue in Nansha, Xiaolou and Zhengguo in Zengcheng, Timian in Huadu, and Lvtian in Conghua.

#### Population distribution

There were 18,396 (49.27%) male and 18,944 (50.73%) female cases reported with a gender ratio 1/1.03 (male/female). The average case age was 39 years old (ranging from one day to 107 years old), while 20–29 year olds accounted for the largest proportion 7972 (21.35%) of cases, followed by 30–39 year olds 6842 (18.32%), 40–49 year olds 6458 (17.30%), 50–59 year olds 5038 (13.49%), 60–69 year olds 3522 (9.43%), 10–19 year olds 2728 (7.31%), 70–79 year olds 2071 (5.55%), 80–89 year olds 1015 (2.72%), 5–9 year olds 928 (2.49%) and <5 age group 766 (2.05%). However, as shown in Additional file 3 and Additional file 4: Table S2, the 80–89 age group had the highest incidence rate (per 100,000 persons) with 554.21, followed by 60–69 group 506.91, 70–79 group 483.39, 50–59 group 403.94, 40–49 group 310.96, 30–39 group 280.33, 20–29 group 237.45, 5–9 group 213.10, 10–19 group187.54 and <5 group 147.18.

With respect to occupation, domestic workers, the unemployed, and retirees accounted for 37.30% (13,926 cases) of all cases, followed by businessmen (11.41%, 4263 cases), workers and employees (11.12%, 4151 cases), students (7.47%, 2792 cases) and farmers (4.89%, 1827 cases).

#### Epidemiological characteristics of imported cases from abroad

As depicted in Fig. 2, a total of 36 foreign imported cases were reported, with 22 laboratory confirmed and 14 clinically diagnosed cases. There was a sharp increase from one to nine cases between April and June, with four cases in May. After June there were no significant changes with two in July, four in August, one in September, five in October, five in November and three in December. Before the first autochthonous case was identified on June 11, there were seven imported cases during May and June from Thailand (four), Malaysia (two), and Saudi Arabia (one), mainly for tourism (six) and business (one).

#### Entomological surveillance

According to the entomological surveillance as depicted in Additional file 5, mosquito density decreased gradually after September 27. BI averaged at 6.47 with max 16.33 before September 27, and averaged 1.90 with max 6.14 after September 27. SSI averaged at 6.71 with max 29.41 before September 27, and averaged 1.16 with max 8.99 after September 27. ADI averaged at 4.12 with max 15.00 before September 27, and averaged at 1.65 with max 7.35 after September 27.

#### Laboratory test

Of the human samples obtained from indigenous cases in 2014, 22,450 (60.12%) underwent laboratory tests in hospitals, including 8337 (37.14%) with NS1 antigen positive and 11,037 (62.86%) with antibody positive (IgM\IgG) for the preliminary screening.

A total of 4589 (12.06%) samples from suspected cases were sent to Guangzhou CDC for IgM and IgG antibody tests. Of them, 2371 (51.67%) were positive, including 672 (14.64%) positive for both IgM and IgG, 1650 (35.96%) positive for IgM alone, and 49 (1.07%) positive for IgG alone. Among the indigenous cases, a total of 106 strains (27.53%) in total 385 sera were isolated, including 96 DENV-1 and 10 DENV-2 strains. Of the 208 RT-PCR positive samples, 198 were positive for DENV-1 and 10 for DENV-2. Among the imported cases, one DENV-2 strain from Thailand was identified (onset time: May 11).

In total of 116 pools of mosquito samples, including 66 larvae and 50 adult, no sample was positive for qRT-PCR.

#### Phylogenetic analysis

**Fig. 4:**
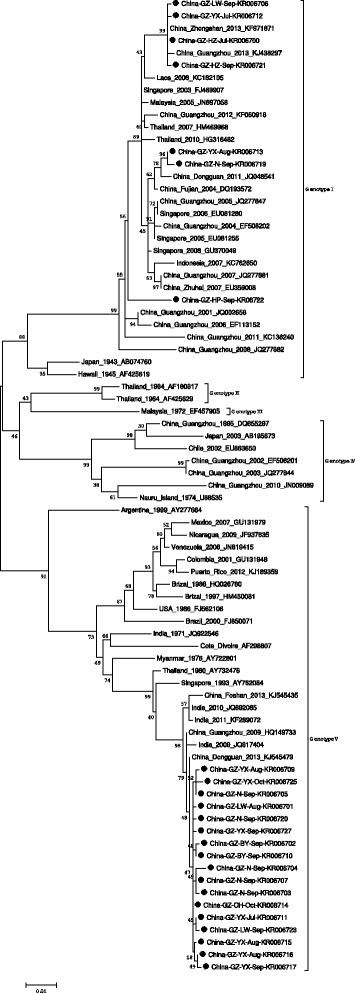
Phylogenetic tree of the DENV-1 E genes isolated in Guangzhou in 2014. 24 sequences from isolated strains and 55 reference sequences from GenBank were aligned using ClustalW. Phylogenetic tree was constructed with the maximum likelihood methods with Kimura 2-parameter corrections of multiple substitutions. Virus strains are indicated by place, date of isolation, and GenBank accession number. Furthermore, the strains isolated in 2014 also were labeled with the district and isolation month

The similarity of the 96 DENV-1 strains was calculated. Sequence with 100% similarity were deleted and one representative sequence was kept. As a result, 24 representative sequences were retained and banked to GenBank with accession no. KR006700–07, KR006709–17, KR006719–23, KR006725, KR006727. Phylogenetic analysis of the E gene from 24 DENV-1 isolates indicated that seven isolates clustered in genotype I and 17 isolates clustered in Genotype V. The genotype V isolates were similar to the isolate isolated in Dongguan City adjacent to Guangzhou in China in 2013 (GenBank accession No. KJ545479), the sample isolated in India in 2009 (GenBank accession No. JQ917404) and isolate from Guangzhou in 2009 (GenBank accession No. HQ149733 imported from Australia). Seven isolates located in genotype I, including four that (GenBank accession No. KR006707, KR006712, KR006700, KR006721) clustered in the same clade with the isolate (GenBank accession No. KJ438297) in 2013 in Guangzhou and the isolate (GenBank accession No. KF971871) in Zhongshan city also adjacent to Guangzhou. Two isolates (GenBank accession No. KR006713, KR006719) showed high similarity with the isolate (GenBank accession No. JQ048541) from Dongguan City in 2011. The final isolate (GenBank accession No. KR06722) clustered in the same parent clade with the genotype I isolates above. See the details in Fig. 4.

**Fig. 5:**
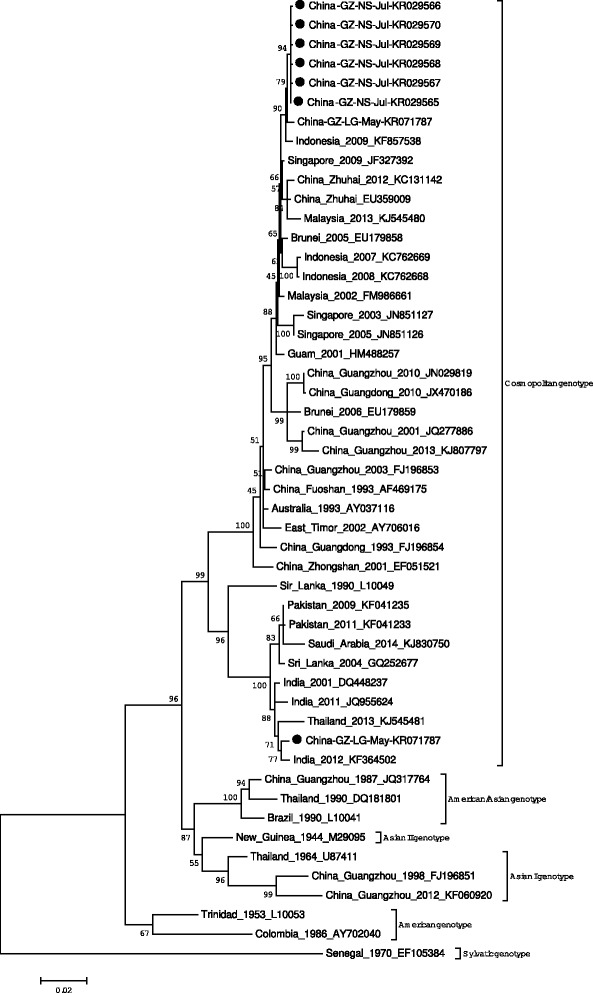
Phylogenetic tree of the DENV-2 E genes isolated in Guangzhou in 2014. Seven sequences from isolated strains and 45 reference sequences from GenBank were aligned using ClustalW. Phylogenetic tree was constructed with the maximum likelihood methods with Kimura 2-parameter corrections of multiple substitutions. Virus strains are indicated by place, date of isolation, and GenBank accession number. Furthermore, the strains isolated in 2014 also were labeled with the district and isolation month

Eleven DENV-2 strains were isolated in 2014. Ten of the strains were from the patients of Dagang community in Nansha District, where scope of the outbreak was limited. Similarity between sequences was calculated, and seven sequences banking in the GenBank with accesssion no. KR029565–70 and KR071787 were phylogenetically analysed. The six strains were similar to the strain isolated in Indonesia in 2009 (GenBank accession No. KF857538) and 2012 (GenBank accession No. KF052653), located in cosmopolitan genotype. Another strain (GenBank accession No. KR071787) came from a patient who came back from Thailand on May 11 in 2014. Compared with the DENV-2 strains isolated in Guangzhou before 2014, the strains are clustered differently. See the details in Fig. 5.

## Discussion

Our results suggested that the unprecedented dengue outbreak in Guangzhou in 2014 was caused by two serotypes of dengue virus, DENV-1 and DENV-2. A similar outbreak of dengue fever had been reported in other cities than Guangzhou of Guangdong Province and in Japan [8]. DENV-2 was isolated in Nansha District only, while DENV-1 was still predominant in Guangzhou.

In the outbreak, the highest incidence rate was observed in domestic workers, the unemployed and retirees. This suggests that patients were probably infected at home or at common sites for exercise or sports of elderly people and retirees. This highlights the importance of outdoor public areas for dengue transmission. Similar findings were reported in the 2014 dengue outbreak in Japan, where many patients were said to be infected in the park [8].

Incidence in persons aged >60 years was larger than that in younger people. This might be due to the fact that young people experienced greater benefits from the success of vector control activities in their households or workplaces. Younger cases also had a greater likelihood of inapparent infections [22], The increase of incidence rate with age in Guangzhou in the 2014 epidemic is in contrast to the higher attack rate in children in traditional endemic areas such as Brazil, Thailand and other Southeastern Asian countries [23]. This might be attributable to the outdoor biting habits of *Aedes albopictus*.

The isolated strains of DENV-1 in 2014 clustered in genotype I and genotype V. The identification and appearance of genotype I provides strong evidence for endemic disease transmission, as it was similar to the strains led to the outbreaks in Guangzhou in 2006 (GenBank accession No. EF113152), 2007 (GenBank accession No. JQ277881) [19], 2011 (GenBank accession No. KC136240) and 2013 (GenBank accession No. KJ438297). It might be the introduction of isolates of genotype I imported from Bangladesh in 2004 (GenBank accession No. EF508202) and 2005 (GenBank accession No. JQ277847) with no indigenous cases were reported. Genotype V also suggested that all isolates in 2014 were similar to isolates collected from Foshan City (GenBank accession No. KJ545435) and Dongguan City (GenBank accession No. KJ545479) in 2013, and the imported isolates (GenBank accession No. HQ149733) from Australia in 2009. It was identified that imported DENV-2 belonging to cosmopolitan genotype in 2014 arouse local ourbreak, due to all the isolates imported abroad after 1987 with not leading to local epidemics.

The mosquito *Aedes aegypti* is the main transmitting vector of dengue, and is distributed in tropical and subtropical regions, however, the principal mosquito for dengue dispersion in Guangzhou is *Aedes albopictus*, with no *Aedes aegypti* identified until 2014 [24, 25]. The density of *Aedes albopictus* increased sharply in the past decade and was highest in 2014, especially in urban areas, which contributed at least in part to the largest outbreak of dengue in Guangzhou. *Aedes albopictus* breeds mostly in the wild and depends on accumulated water in various containers or plants. As the subtropical geographical environment and climate in Guangzhou is similar to Southeastern Asia where *Aedes aegypti* is the main vector for dengue, Guangzhou’s rainfall and air temperature is suitable for the growth and reproduction of *Aedes albopictus* both indoors and outdoors*.* Additionally, local customs among residents of breeding *Dracaena sanderiana* and *Rohdea japonica* provides a variety of suitable breeding sites for mosquito vectors.

Generally, dengue epidemics transmitted by *Aedes albopictus* tend to be mild and short-lived [26], such as the epidemics that occurred in Henan in China [27], France, Croatia [13], Japan [8] and North America [28]. However, recent studies indicated that the propagation efficiency of *Aedes albopictus* for DENV transmission was as high as that of *Aedes aegypti*, and *Aedes albopictus* infected with DENV show higher concentrations of DENV RNA in abdominal tissues compared to *Aedes aegypti* [29]. It is likely that the increased intensity of transmission and final outbreak size of the 2014 Guangzhou epidemic were in part due to entomological characteristics of *Aedes albopictus*. These findings suggest the potnential for large outbreaks in areas such as Europe and the United States which are endemic for *Aedes albopictus* [10]. Song et al. [30] found apparent lagged effects on the relationship between mosquito abundance and dengue fever, and the lag time was no more than two months in most years, which was consistent with the incubation periods of dengue. Our study also showed cases peaked about one month after peak adult mosquito densities, in line with these findings.

As a main epidemic center, the attributes of Guangzhou represents the future trend in mainland China. There has been much recent debate on the endemic status of dengue in China among government and academic institutions, including the capacity for local transmission versus the importance of importation. The critical factors for local transmission in Guangzhou are imported cases and mosquito density [30]. The number of imported cases is a strong determinant of final outbreak size, which was confirmed in 2010 by epidemiological and phylogeographic methods [17]. In this study, we deem the dengue fever caused by DENV-2 as imported [31], while that caused by DENV-1 appeared to be endemic disease. Therefore, the Guangzhou government should strengthen vector control measures and increase awareness among residents to prevent similar outbreaks.

The major limitation in this study is that no dengue virus was identified in the larval or adult mosquito samples we collected. In addition, due to the scarcity of isolates from imported cases for DENV-1, the judgment of imported or indigenous epidemic warrants further investigation. Urgent future work should include deep analysis on local vector and host interactions and their capacity to result in large-scale outbreaks.

## Conclusions

The record-breaking dengue outbreak in 2014 was confirmed as a first-time co-circulation of DENV-1 and DENV-2 in Guangzhou, warning a high efficient transmission by *Aedes albopictus*. The DENV-1 epidemic has been already an endemic disease, while the DENV-2 epidemic was imported.

## Additional files

Additional file 1: Multilingual abstract in the five official working languages of the United Nations. (PDF 689 kb)

Additional file 2: Table S1. The district distibution of dengue outbreak in Guangzhou, 2014. (XLS 25 kb)

Additional file 3: The age distribution of dengue outbreak in Guangzhou, 2014. The brown bar chart shows the age-specific cumulative cases and the dark red line represents the incidence rate of different age groups. (XLS 400 kb)

Additional file 4: Table S2. The Age district distibution of dengue outbreak in Guangzhou, 2014. (TIFF 83 kb)

Additional file 5: Daily entomological surveillance in dengue outbreak in Guangzhou, 2014. The cyan line depicts the daily mean *Breteau Index* (BI), with the lawngreen line of *Standard Space Index* (SSI) and blueviolet line of *Adult Mosquitos Density Index* (ADI). The transparent background shows the epicurve and report curve. (TIFF 227 kb)

## Abbreviations

ADI: *Adult Mosquito Density Index*
BI: *Breteau Index*
DENV: Dengue virus
DHF: Dengue hemorrhagic fever
DSS: Dengue shock syndrome
ELISA: Enzyme linked immunosorbent assay
GZCDC: Guangzhou Center for Diseases Control and Prevention
ICGT: Immune colloidal gold technique
NIDRS: Notifiable Infectious Disease Report System
NS1: Nonstructural protein 1
PCR: Polymerase Chain Reaction
SSI: *Standard Space Index*
WHO: World Health Organization
