# Supplementary material for: The dengue preface to endemic in mainland China: the historical largest outbreak by Aedes albopictus in Guangzhou, 2014
Source: Infect Dis Poverty. 2017 Sep 22;6:148. doi: 10.1186/s40249-017-0352-9 (PMC5609019; doi:10.1186/s40249-017-0352-9)
Supplement: Supplementary file 1 — Multilingual abstract in the five official working languages of the United Nations. (PDF 689 kb) [file 40249_2017_352_MOESM1_ESM.pdf]

Translation of the abstract into the five official working languages of the United Nations

بدء توطن حمي الضنك في أراضي الصين: أكبر تفشي تاريخي للمرض بواسطة بعوض الزاعجة المنقطة بالأبيض في قوانغتشو في 2014

لي لو, لي يون جيانغ, شين-تساي شياو, بيو دي, تشين لونغ جينغ, شنغ-يونغ وانغ, جين لينغ تانغ, مينغ وانغ, شياو بينغ تانغ, تشي كونغ يانغ

الملخص

الخلفية: في السابق تم إعتبار حمي الضنك وباءا خفيفا في الصين ينتقل بواسطة بعوض الزاعجة المنقطة بالأبيض ولكن حدوث أكبر تفشي تاريخي للمرض في قوانغتشو سنة 2014 قد يغير الوضع ومن أجل التعرف علي الاتجاه الوبائي وتقديم الأدلة لاستراتيجيات وتدابير الوقاية والمكافحة, تنتقل حمي الضنك في الصين بواسطة بعوض الزاعجة المنقطة بالأبيض وتم إهمال المرض تاريخيا وإعتباره من الامراض المعدية ذات الأهمية الثانوية ولكن تفشي المرض مؤخرا في قوانغتشو يمكن ان يغير الوضع نظرا لضخامة التفشي وبهدف توفير إنذار مبكر للاتجاهات الوبائية وتقديم الأدلة لاستراتيجيات الوقاية والمكافحة فإننا نسعى إلى توصيف تفشي الوباء من خلال تطبيق البيانات التفصيلية المتعلقة بحالات الإصابة بالمرض وانتشار الحشرات الناقلة له وكذلك تحليل تطور سلالات الفيروسات المسببة للمرض.

الأساليب: تم إستخدام بيانات فحص الحالات التي تم تحديدها من خلال نظام الإبلاغ عن الامراض المعدية القابل للإخطار ومراقبة الحشرات والفحص السيرولوجي للسكان إلى جانب اختبار مختبري للغلوبولين المناعي إم/جيم والبروتين الغير تركيبي ن س 1 وعزل العينات الفيروسية وتحليل التتابع الجيني للجين المغلف لها وتحليلات تطور سلالات الفيروس لدراسة الخصائص الوبائية والجزيئية لهذا الفاشية.

النتائج: تفشي مرض حمي الضنك سنة 2014 في قوانغتشو ليصل لحوالي 80% من إجمالي الحالات المبلغ عنها في تلك السنه في الصين وتم الإبلاغ عن ما مجموعه 37376 حاله من بينها 37340 حاله من حالات السكان الأصليين بلغت نسبه الإصابة بها 2908.3 لكل مليون و 36 حاله تم دخولهم الي قوانغتشو مع وضع عدد 14055 حالة في المستشفيات وحدوث 5 حالات وفاه واستمر الوباء لمده 193 يوما في الفترة من 11 يونيو إلى 21 ديسمبر وكانت أعلي نسبة إصابة تم ملاحظتها في العاملين في المنازل والعاطلين عن العمل والمتقاعدين وكان معدل الإصابة غير الظاهر 18.00 ٪ (750/135) وفي المجموع تم عزل 96 سلالة من فيروس حمي الضنك 1 (DENV-1) و 11 سلالة من فيروس حمي الضنك 2 (DENV-2) وأشار تحليل تطور السلالات إلى ان السلالات التي كانت من طراز "DENV-1" تنقسم إلى النمط الجيني الأول والخامس وهو ما يماثل السلالات المعزولة في قوانغتشو ودونغ غوان في 2013 وكانت السلالات من طراز "DENV-2" مماثله لتلك التي وردت من تايلند في 11 مايو في 2014 والتي وردت من اندونيسيا في 2012.

الاستنتاجات: تم التأكيد على ان وباء حمي الضنك الذي تفشي في 2014 هو أول مره يتم فيها التداول المزدوج لكل من طرازي الفيروس "DENV-1" و "DENV-2" في قوانغتشو وكانت سلاله "DENV-1" متوطنه بينما كانت سلاله "DENV-2" واردة من الخارج وتنتقل بكفاءة بواسطة بعوض الزاعجة المنقطة بالأبيض بمستويات عالية مثل تلك التي يسببها بعوض الزاعجة المصرية.

Translated from English version into Arabic by Mohamed Habib

登革热在中国大陆的流行: 2014 年广州发生历史上最大规模的由白纹伊蚊引起的暴发

罗雷，蒋力云，肖新才，狄飙，景钦隆，王声涌，唐金陵，王鸣，唐小平，杨智聪

摘要

引言：在中国大陆，登革热的流行一直被认为是由白纹伊蚊引起的轻度流行。然而，2014 年广州历史上发生最大规模的暴发改变了这一流行特征。为提供疫情早期流行的预警，制定针对性防控策略，笔者利用详细的病例信息、虫媒监测信息和 E 基因进化树分析总结 2014 年登革热暴发流行的特点。

方法：本研究使用传染病报告管理系统中的确诊病例、蚊媒监测数据、人群血清学调查数据和血标本 IgM/IgG、NS1 检测和 E 基因测序等实验室检测信息分析 2014 年广州登革热的流行病学和分子生物学特征。

结果：2014 年广州登革热报告病例数占全大陆报告病例数约 80%；全年共报告 37376 例登革热病例，其中 37340 例本地病例，本地病例发病率为 290.83/10 万；共导致 14055 例病例住院和 5 例死亡。整个流行期从 6 月 11 日至 12 月 21 日共持续 193 天；发病人群主要为家居人群、失业或退休者。人群血清学监测隐性感染率为 18.00%（135/750）。共分离出 96 株 DENV-1 病毒和 11 株 DENV-2 型病毒。系统进化分析显示 DENV-1 型的基因型为 I 和 V，与 2013 年广州和东莞分离株的基因型相似。DENV-2 型与泰国 2014 年和印度尼西亚 2012

年分离株相同。
结论：2014 年广州登革热流行被证实为 DENV-1 和 DENV-2 引发的共同流行，其中 DENV-1 为主导流行血清型，DENV-2 型主要以输入为主。广州登革热的流行提示白纹伊蚊与埃及伊蚊有同等高效的登革病毒传播效率。

Translated from English version into Arabic by Lei Luo

Montée endémique de la dengue dans la Chine continentale: la plus grande épidémie du temps par A*edes albopictu*s à Guangzhou, 2014

Lei Luo, Li-Yun Jiang, Xin-Cai Xiao, Biao Di, Qin-Long Jing, Sheng-Yong Wang, Jin-Ling Tang, Ming Wang, Xiao-Ping Tang and Zhi-Cong Yang

Résumé
Contexte: L'épidémie de dengue, dûe à *Aedes albopictus* a été considérée comme modérée dans la Chine continentale. Toutefois, la plus importante flambée historique de 2014 à Guangzhou laisse changer de perception. Afin de fournir une alerte rapide des tendances épidémiques et des preuves pour les stratégies de prévention et de contrôle, nous avons cherché à définir le foyer de 2014 en faisant recours aux détails des cas et aux données entomologiques, ainsi qu'à l'analyse phylogénétique du gène (E) de l'enveloppe virale.
Méthodes: Nous avons utilisé les données d'étude de cas identifiées par le Système de Surveillance des Maladies Infectieuses à Déclaration Obligatoire, la surveillance entomologique et l'étude sérologiqque de population, ainsi que des tests de détection d'anticorps IgM/IgG ou d'antigènes NS1, et l'isolement des échantillons viraux suivi du séquençage du gène E et de l'analyse phylogénétique afin déterminer les caractéristiques moléculaires et épidémiologiques de l'épidémie.
Résultats: Le nombre de cas lors de cette épidémie de la fièvre dengue de 2014 à Guangzhou représentait près de 80% du nombre total de cas signalés cette année-là dans la Chine continentale; un total de 37 376 cas y compris 37 340 cas autochtones avec un taux d'incidence de 2 908.3 par million et 36 cas importés ont été signalés à Guangzhou, avec 14 055 cas d'hospitalisation et cinq décès. L'épidémie a duré 193 jours du 11 Juin au 21 Décembre avec la plus forte incidence observée chez les travailleurs domestiques, les chômeurs et les retraités. Le taux d'infection asymptomatique était de 18% (135/750). Au total, quatre-vingt-dix échantillons de la souche sérotype 1 du virus de la fièvre dengue (DENV-1) et onze échantillons de la souche sérotype 2 (DENV-2) ont été isolés. L'analyse phylogénétique a indiqué que les souches DENV-1 étaient divisées en génotype I et V, semblables aux souches isolées à Guangzhou et Dongguan en 2013. Les souches DENV-2 étaient similaires à celles importés de Thaïlande le 11 Mai 2014 et aussi à celles importées d'Indonésie en 2012.
Conclusion: L'épidémie de dengue de 2014 a été confirmée d'être la première à avoir propagé la double infection aux souches DENV-1 et DENV-2 à Guangzhou. La souche DENV-1 était endémique, tandis que la souche DENV-2 a été importée, tout en étant efficacement transmis par l'espèce *Aedes albopictus* à des niveaux d'infection aussi élevés que par le vecteur *Aedes aegypti*.

Translated from English version into French by Kokouvi Kassegne

Dengue начинало превратиться в эндемическое заболевание в Китае: историческая крупнейшая вспышка в связи с *Ades albopictus* в Гуанчжоу, 2014

Lei Luo, Li-Yun Jiang, Xin-Cai Xiao, Biao Di, Qin-Long Jing, Sheng-Yong Wang, Jin-Ling Tang, Ming Wang, Xiao-Ping Tang and Zhi-Cong Yang

Реферат
Введение: Dengue рассматривалось в качестве мягкого эпидемии в Китае, передаваемых может изменить ситуацию. Для того, чтобы распознать тренд эпидемии и предоставить доказательства в стратегии профилактики и контроля и меры. Исторически сложилось так,

что Dengue в Китае, переданное комаров-переносчиками *Aedes albopictus* и игнорировалось как инфекционное заболевание второстепенного значения. Однако, недавняя вспышка 2014 в Гуанчжоу исторического масштаба может изменить ситуацию. Для того, чтобы оказывать раннее предупреждение о тенденциях развития эпидемии и предлогать доказательства для профилактики и стратегии управления мы стремимся, чтобы характеризовать вспышка 2014 по применению подробных дел и энтомологических данных, а также анализа вирусной филогении.
Методы: Мы использовали данные исследования выявлять через Notifiable Infectious Disease с испытательной лабораторией по IgM/IgG, а также NS1, и изоляции вирусных образцов с последующим секвенированием генов конверта и филогенетического анализа, чтобы изучить эпидемиологические и молекулярные характеристики по этой вспышке.

Результаты: Вспышка 2014 в Гуанчжоу приходится почти 80% всех зарегистрированных случаев того года в Китае; в общей сложности 37376 случаи, включая 37340 местных случаев заболеваемости с 2908.3 на миллион и 36 завезенных случаев заболевания были зарегистрированы в городе Гуанчжоу, в том числе и 14055 человек госпитализированы и 5 погибли. Эпидемия продолжалась в течение 193 дней с 11 июня по 21 декабря, самый высокий уровень заболеваемости наблюдается в отечественных рабочих, безработных и пенсионерах. Ставка инфекции inapparent составляет 18.00% (135/750). В общей сложности девяносто шесть вируса dengue 1 (DENV-1) и 11 вирусов dengue 2 (DENV-2) штаммы были изолированы. Филогенетический анализ показал, что DENV-1 штаммы были разделены на Генотип I и V, похожие на штаммы, которые были выделенны в Гуанчжоу и Дунгуань в 2013 году. DENV-2 штаммы были аналогичны тем, которые импортированы из Таиланда 11 мая 2014 года и импортировались из Индонезии в 2012 году.
Заключение: Эпидемия dengue 2014 было подтверждено представлять собой первое появление совместного обращения DENV-1 и DENV-2 в Гуанчжоу. DENV-1 штамм был эндемическим заболеванием, когда DENV-2 штамм был ввезен и эффективно передаваемый комарами *Aedes albopictus* с векторной видов на высоких уровнях, как *Aedes aegypti*.

Translated from English version into French by Hao-Qi Zhang

El prefacio del dengue a endémico en China continental: El brote más grande histórico de Aedes albopictus en Guangzhou, 2014

Lei Luo, Li-Yun Jiang, Xin-Cai Xiao, Biao Di, Qin-Long Jing, Sheng-Yong Wang, Jin-Ling Tang, Ming Wang, Xiao-Ping Tang and Zhi-Cong Yang

Abstracto

Trasfondo: El dengue fue considerado como una epidemia leve en China continental transmitida por *Aedes albopictus*. Sin embargo, el mayor brote histórico en 2014 en Guangzhou podría cambiar la situación. Con el fin de reconocer la tendencia epidémica y proporcionar evidencia de estrategias y medidas de prevención y control. Históricamente, el dengue en China continental, transmitido por el mosquito vector Aedes albopictus ha sido descuidado como una enfermedad infecciosa de importancia secundaria. Sin embargo, el reciente brote de 2014 en Guangzhou de magnitud histórica podría cambiar la situación. Con el fin de proporcionar una alerta temprana de las tendencias de la epidemia y proporcionar pruebas para la prevención y estrategias de control, nosotros buscamos caracterizar el brote 2014 a través de la aplicación de casos detallados y datos entomológicos, así como el análisis de filogenias virales.
Métodos: Se utilizaron datos de encuestas de casos identificados a través del Sistema de Notificación de Enfermedades Infecciosas, vigilancia entomológica y sero-encuestas de población, junto con pruebas de laboratorio para IgM / IgG, NS1 y aislamiento de muestras virales seguido de secuenciación genética de envoltura y análisis filogenéticos para examinar las características epidemiológicas y moleculares características de este brote.
Resultados: El brote de dengue de 2014 en Guangzhou representó casi el 80% del total de casos notificados ese año en China continental; Un total de 37,376 casos, incluidos 37,340 casos nativos con una tasa de incidencia de 2,908.3 por millón y 36 casos importados fueron reportados en

Guangzhou, 14,055 hospitalizados y 5 muertes. La epidemia duró 193 días, desde el 11 de junio hasta el 21 de diciembre, con la mayor incidencia observada en los trabajadores domésticos, los desempleados y los jubilados. La tasa de infección inaparente fue de 18.00% (135/750). En total, se aislaron noventa y seis cepas del virus del dengue 1 (DENV-1) y 11 cepas del virus del dengue 2 (DENV-2). El análisis filogenético indicó que las cepas DENV-1 se dividieron en los genotipos I y V, similares a las cepas aisladas en Guangzhou y Dongguan en 2013. Las cepas DENV-2 aisladas fueron similares a las importadas de Tailandia el 11 de mayo de 2014 y que se importaron desde Indonesia en 2012.
Conclusiones: La epidemia de dengue de 2014 fue confirmada como la primera ocurrencia de co-circulación de DENV-1 y DENV-2 en Guangzhou. La cepa DENV-1 fue endémica, mientras que la cepa DENV-2 fue importada, siendo eficientemente transmitida por la especie vectorial *Aedes albopictus* a niveles tan altos como *Aedes aegypti*.

Translated from English version into French by Laura C Vicente Rodriguez
