# Supplementary figures and images for: The dengue preface to endemic in mainland China: the historical largest outbreak by Aedes albopictus in Guangzhou, 2014
Source: Infect Dis Poverty. 2017 Sep 22;6:148. doi: 10.1186/s40249-017-0352-9 (PMC5609019; doi:10.1186/s40249-017-0352-9)

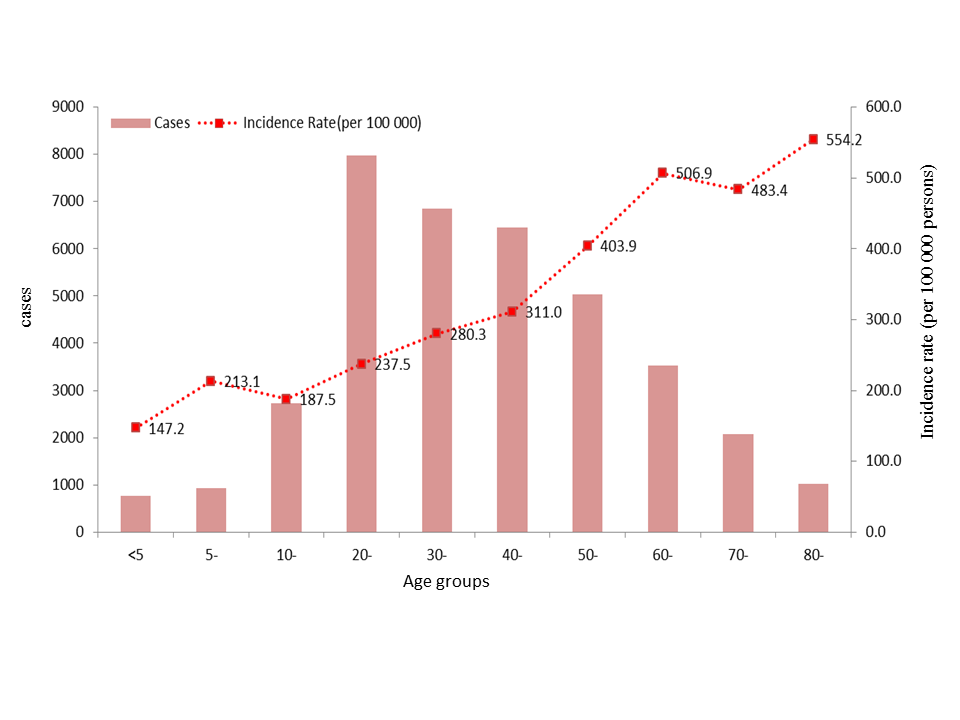

Supplement: Supplementary file 4 — The Age district distibution of dengue outbreak in Guangzhou, 2014. (TIFF 83 kb) [file 40249_2017_352_MOESM4_ESM.tif]

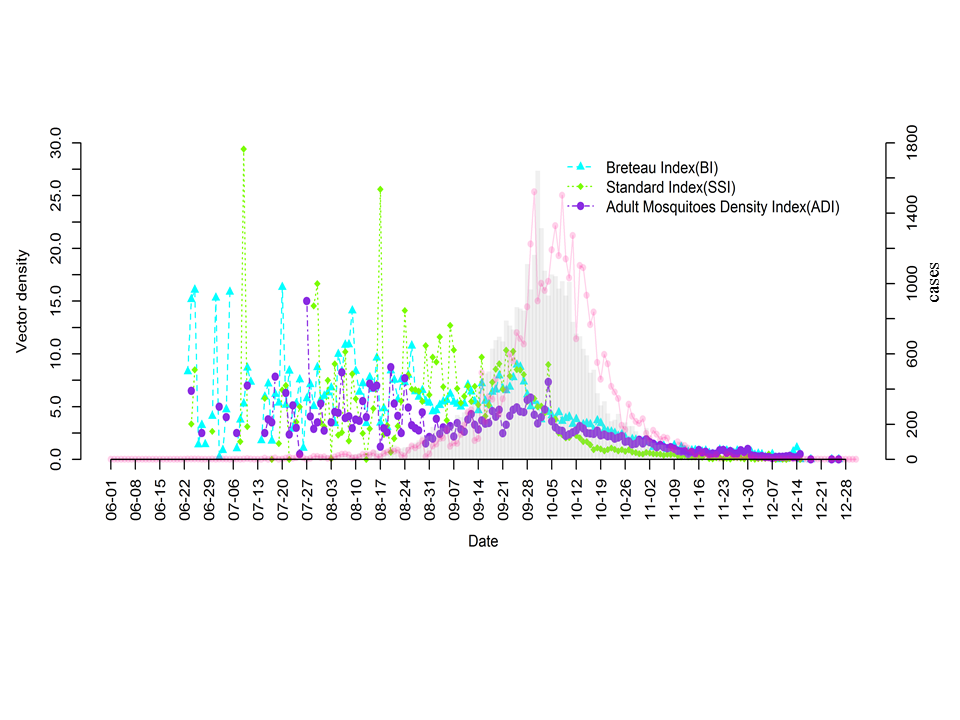

Supplement: Supplementary file 5 — Daily entomological surveillance in dengue outbreak in Guangzhou, 2014. The cyan line depicts the daily mean Breteau Index (BI), with the lawngreen line of Standard Space Index (SSI) and blueviolet line of Adult Mosquitos Density Index (ADI). The transparent background shows the epicurve and report curve. (TIFF 227 kb) [file 40249_2017_352_MOESM5_ESM.tif]
